# Update on Radiation Safety in the Cath Lab – Moving Toward a “Lead-Free” Environment

**DOI:** 10.1016/j.jscai.2023.101040

**Published:** 2023-06-02

**Authors:** Ariel Roguin, Perry Wu, Travis Cohoon, Fahad Gul, George Nasr, Ned Premyodhin, Morton J. Kern

**Affiliations:** a Department of Cardiology, Hillel Yaffe Medical Center, Hadera, Israel; b Division of Cardiology, University of California – Irvine, Irvine, California; c VA Long Beach, Long Beach, California; d Faculty of Medicine, Technion - Israel Institute of Technology, Israel

**Keywords:** coronary angiography, coronary intervention, radiation

## Abstract

Radiation exposure in the cardiac catheterization laboratory (CCL) is an occupational hazard that predisposes health care workers to the development of adverse health effects such as cataracts, cancer, and orthopedic injury. To mitigate radiation exposure, personal protective shielding as well as permanently installed shields reduces these adverse effects. Yet, heavy protective lead aprons and poor ergonomics required for positioning movable shields remain barriers to a safer environment. Recent innovations to enhance personal protective equipment and revolutionize fixed shielding systems will permit the CCL team to work in a personal “lead-free” environment, markedly reducing occupational hazards. The purpose of this review is to update the status and future of radiation protection in the CCL.

## Highlights

• Heavy protective lead aprons, moveable shields, and poor ergonomics remain barriers to a safer environment.
• Innovations in shielding systems will permit the cardiac catheterization laboratory team to work in a personal “lead-free” environment.
• Improving radiation protection will enhance the quality of life for the cardiac catheterization laboratory team .

## Central Illustration

**Figure:**
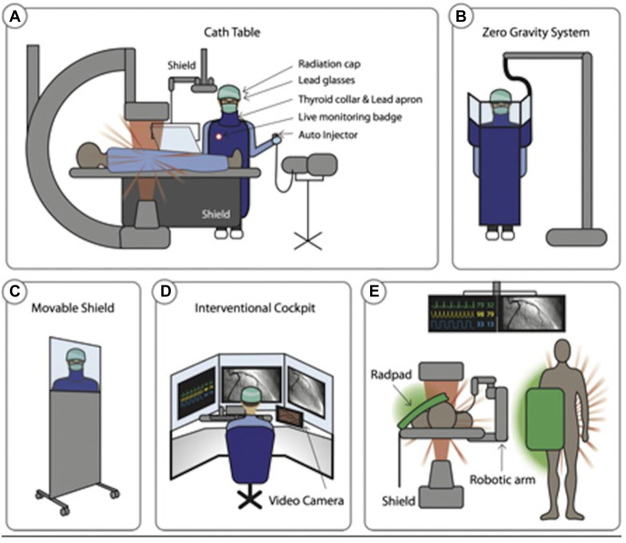

## Introduction

With advancements in percutaneous transcatheter interventions, complex procedures can become prolonged with increased radiation exposure to the proceduralist and his/her team. Advanced interventions with high degree of radiation exposure include multivessel percutaneous coronary intervention (PCI), particularly high-risk PCI, chronic total occlusion (CTO) PCI, transcatheter aortic valve replacement, and transcatheter edge-to-edge repair of the mitral valve and tricuspid valve interventions.^1^, ^2^, ^3^, ^4^, ^5^, ^6^ As a rule, obtaining quality images to optimize interventions should not necessitate increasing the ordinary procedural radiation exposure to the lab personnel. In 2020, the Society for Cardiovascular Angiography & Interventions published a multi-society position statement calling for strategies to mitigate radiation-related hazards inherent to the fluoroscopic laboratory.^7^ Application of best practices to enhance radiation safety will reduce the exposure to patient, operators, and the cardiac catheterization laboratory (CCL) team.

All CCL procedures should be performed with the goal of acquiring the necessary clinical information while keeping radiation doses as low as reasonably achievable and minimizing exposure to health care workers. The implementation of novel radiation shielding technology aims to permit operators to work in a personal protective “lead-free” (ie, without wearing lead aprons, etc) environment.

### Established best practices in personal protective equipment and shielding

The current practice requires all members of the CCL team wear personal protective equipment with at least lead body aprons and thyroid shields. Some operators also use leaded skull covering, leaded eyeglasses, and arm shields. Conventional lead aprons are now being replaced with newer aprons made of lighter materials that include aluminum, antimony, barium, bismuth, tungsten, tin, and titanium. Some of these materials may reduce personal protective apron weight by 20% to 40%.^8^ Yet, these composite aprons may still pose significant cumulative orthopedic burden to the members of the CCL team. Moreover, many composite garments did not meet manufacturer-stated lead equivalence when attenuation was tested under scattered radiation.^9^ A protective apron lead thickness standard of 0.5 mm should stop 95% of scatter radiation, and protective integrity should be checked regularly.^10^

Every CCL should have a ceiling-mounted, movable upper-body shield and lower-body shield mounted on the side of the patient table.^9^ A ceiling-suspended screen and a curtain shield under the table reduce scatter radiation by approximately 80% to 90%.^11^^,^^12^ The protective shielding should form a continuous “curtain” between the operator and the radiation source. Portable lead acrylic mobile shields can be used to further protect the nursing and ancillary staff. These shields can be placed in proximity to the patient to increase protection during prolonged fluoroscopy procedures such as electrophysiology (EP) procedures or structural heart or CTO coronary interventions.

#### Health care workers at highest radiation exposure risk

**Figure 1:**
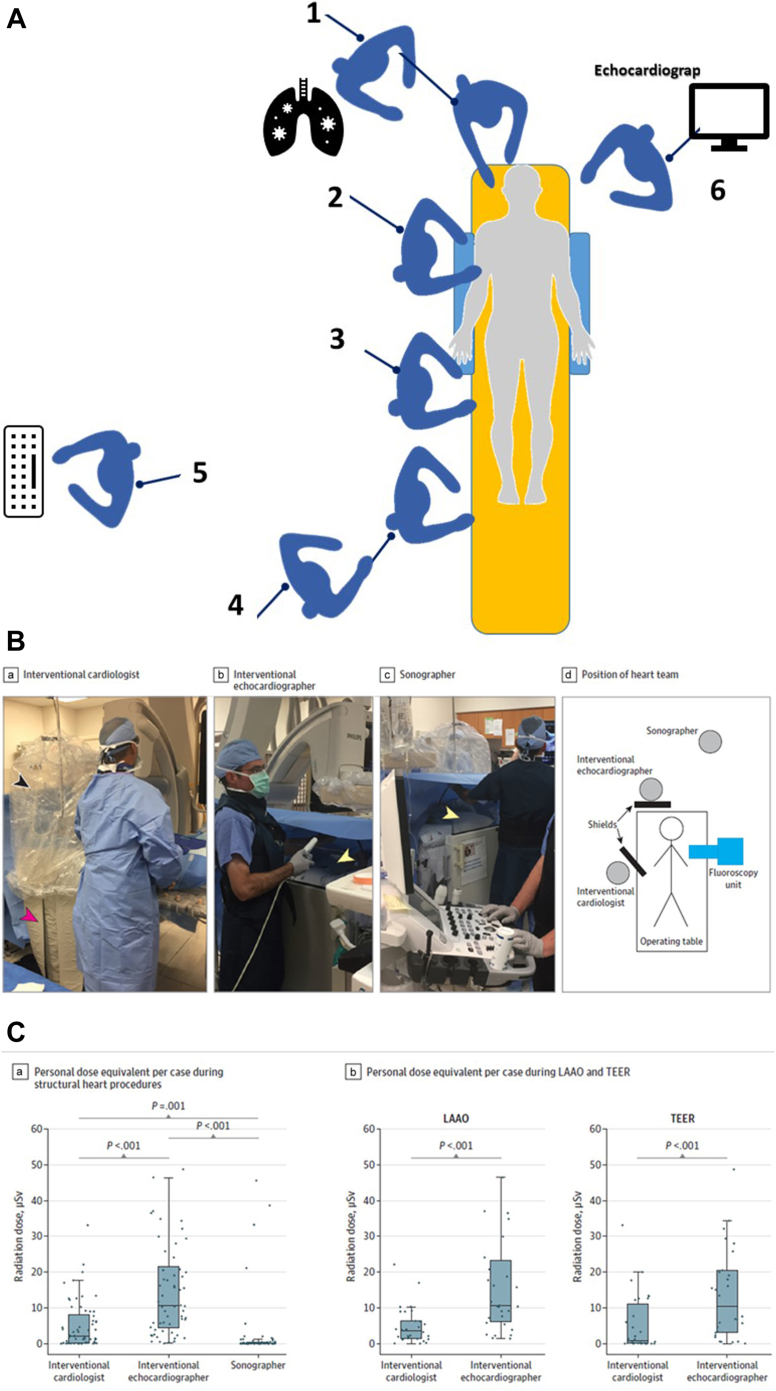
(**A**) **Diagram of locations which are exposed to radiation in the cath lab.**Compared to the pre-structural heart disease era, there are now personnel located closer to the x-ray tube for anesthesia and transesophageal echocardiography operations. The personnel located closer to the x-ray tube for anesthesia and TEE operations. 1, respiratory therapist and anesthesiologist, 2 operator, 3 operator assistant, 4, circulating nurses, 5 recording technician/nurse, 6 echocardiographer. Courtesy of Dr. Robert Wilson. (**B**) Left atrial appendage closure and transcatheter edge-to-edge mitral valve repair were performed with an interventional cardiologist standing immediately adjacent to the procedure table. (a)Interventional cardiologists used a ceiling-mounted upper-body lead shield (black arrow) and a lower-body lead shield attached to the side of the operating table extending from the table to the floor (red arrow). (b) The interventional echocardiographer stood at the patient’s head to manipulate the transesophageal echocardiogram probe during the procedure. Interventional echocardiographers used a mobile, height-adjustable, accessory lead shield (yellow arrow). The upper section of the shield was raised to a height that allowed the interventional echocardiographer to extend their arms over the shield to manipulate the transesophageal echocardiogram probe throughout the case. (c) A sonographer (right), who assisted with image acquisition throughout the procedure, stood approximately 250 cm from the radiation source. The nearness of the interventional echocardiographer to the radiation source is evident in this image. (d) Overhead diagram shows the relative position of the interventional cardiologist, interventional echocardiographer, and sonographer to the patient. From McNamara et al.^13^ (**C**) Radiation doses during structural heart procedures. (a) Distribution of personal dose equivalent per case for interventional cardiologists, interventional echocardiographers, and sonographers during structural heart procedures (n = 60). (b) Distribution of personal dose equivalent per case for the interventional cardiologist and interventional echocardiographer during percutaneous left atrial appendage occlusion (LAAO) (n = 30) and percutaneous transcatheter edge-to-edge mitral valve repair (TEER) (n = 30) are shown. From McNamara et al.^13^

In the era of structural heart disease and complex EP or coronary interventional procedures, personnel at risk include not only the patient and primary operators but also radiology technicians, nursing staff, ancillary physicians such as anesthesiologists and echocardiographers as well as device representatives.^13^ The positions of these team members around the x-ray table often determine the exposure risk (Figure 1).

An interventional cardiologist’s radiation exposure varies with the length and complexity of the procedure, patient characteristics, and radiation protection equipment available.^14^ When compared to staff interventional cardiologists, fellows-in-training are at higher risk of radiation exposure.^15^ When adjusting for complexity, a prospective trial found fellows-in-training were exposed to 34% more radiation than staff interventionalists. This finding is likely attributable to fellows’ eagerness to learn and to place patient safety over their own, failure to optimize collimation settings, and acquisition of more fluoroscopy recordings than necessary.^15^

Studies have shown anesthesiologists to be at highest risk of radiation exposure, which is likely attributable to ineffective shielding during procedures.^16^ Despite being near equal distances to the C-arm, scrub nurses and radiation technicians received 1/15th the amount of radiation compared to anesthesiologists due to adequate shielding and positioning behind the primary operator.^16^

#### Special populations at risk

A special population at risk is pregnant women. Radiation exposure can have deleterious effects on the fetus including delayed mental development, intrauterine growth retardation, and organ malformation. There is no difference in fetal outcomes in women exposed to a cumulative radiation dose <50 mGy during their pregnancy when compared to the general population. Consequently, the National Council on Radiation Protection and Measurements has limited cumulative exposure to 0.5 mSV per month or to a total of 5 mSv during the span of the pregnancy.^17^^,^^18^ During most PCIs, the patient may receive a total of 8 to 10 mSv of radiation. However, more complex procedures and increased patient body mass index may increase cumulative radiation exposure.^19^

EP procedures may give patients an effective dose of 6.6 to 59.7 mSV for atrial fibrillation ablation and up to 95 mSv for cardiac resynchronization therapy implantations. This compares to up to 16 mSv for diagnostic coronary angiograms and 57 mSv for PCI and structural procedures.^16^ Electrophysiologists are also at increased risk of radiation exposure compared to other interventionists, particularly during implantation of cardiac pacing and other similar devices.^20^ In these cases, factors that contribute to increased exposure include positioning of the operator on the left side of the patient with lack of shielding to accommodate these procedures, longer fluoroscopy time with cardiac resynchronization device implants, and unprotected scatter from the patient.

CCL staff should be routinely re-educated about radiation safety, including considerations for pregnant patients and staff. Staff should be encouraged to advocate for appropriate protective equipment or implement systems changes to meet safety standards. Best practices in the CCL are provided in Table 1 along with advanced practices and enhancements in each of these categories.

**Table 1:** Best practices in the cardiac catheterization laboratory with associated advanced practices and enhancements.

Current practices		Advanced practices	Benefits of novel technology
Accessory Personal protective equipment (PPE)	Dosimeter badges	- RaySafe i2 system	Real-time dose detection
	Thyroid shield: standard nominal thickness of 0.25 to 0.5 mm of lead, with effective shielding area ∼300 cm^2^	- Tight fit of thyroid collar around neck - Bismuth masking reagent	Reduce risk of thyroid cancers
	Leaded glasses: optimal thickness of 0.35 mm to 0.5 mm of lead	- Proper fit and facial contour. - Reduce gap between lens and frame - Maximize front, lateral, and angular protection	Reduce rates of cataracts
Operator techniques	Reduced fluoroscopy intensity or time	- Decreasing frame rate - Fluoro-save	Minimize cine usage
	Collimation	- Automatic dose rate control (ADRC) - ControlRad	Precise collimation of area of interest
	Avoid magnification	- Live Zoom	Decrease magnification which increases radiation
	Distance to table	Tubing extensions on contrast injectors	Allows operator to stay further away during imaging
Environmental radiation protection	Ceiling-mounted upper-body radiation shields and movable table-mounted curtain shields	- RADIACTION - Patient Pelvic shielding - RADPAD - EggNest-XR system - Steradian vertical radiation shield	Greatly reduce scatter to the operator and the rest of the team from both above the table and under the table.
	Lead aprons and portable radiation shields	- Zero-Gravity system - Rampart M1128 - Protego Radiation Protection System - Corindus CorPath robotic system	Reduces radiation drastically with large barrier device, allowing operators and/or most of the CCL team to work “lead-free”

#### Radiation monitoring

**Figure 2:**
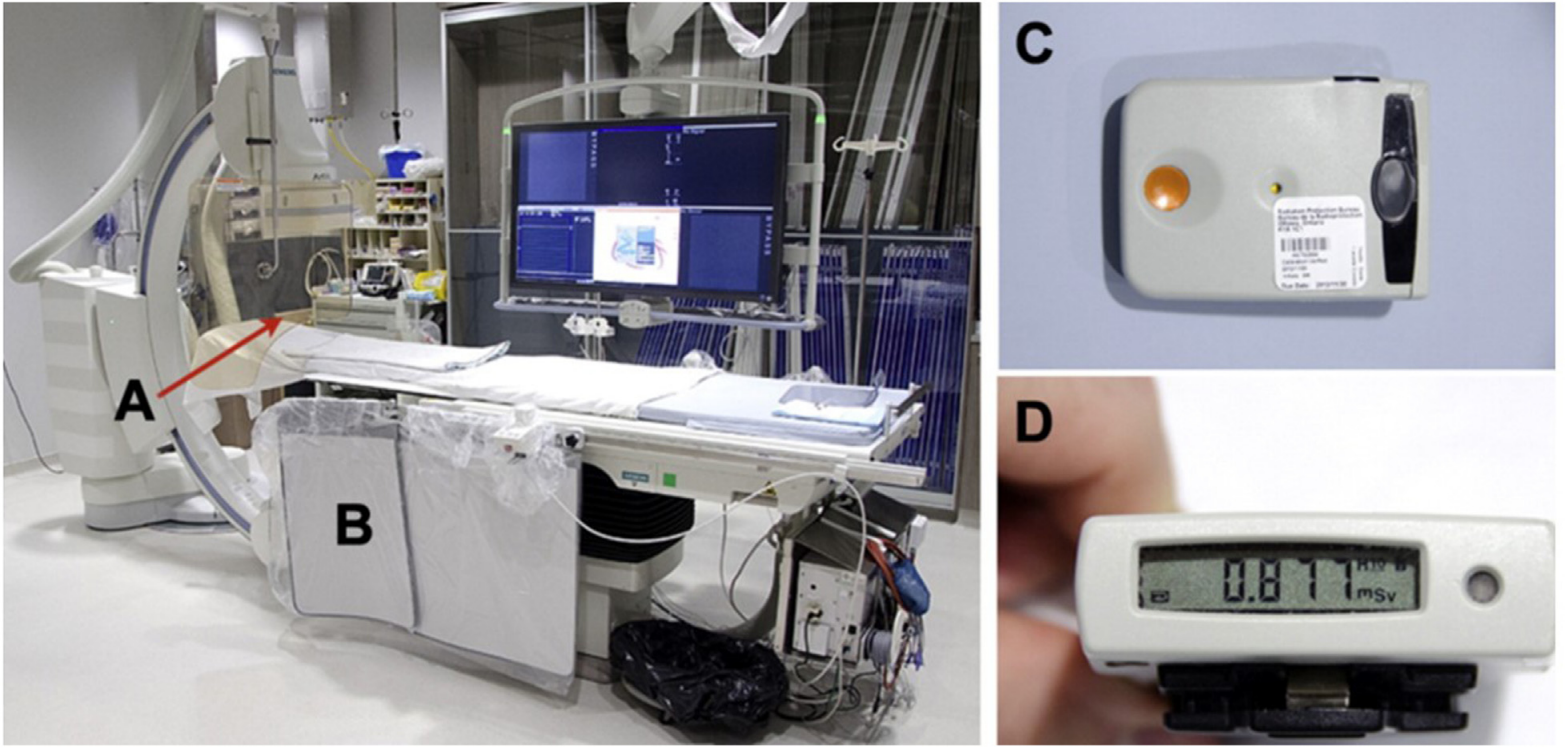
**Novel RaySafe i2 system offers real-time x-ray radiation dose monitoring.** (**A**) Ceiling-suspended acrylic shielding (1-mm lead equivalent), and (**B**) table-to-floor lead flap. (**C**) Electronic personal dosimeters worn by operators. An electronic personal dosimeter was worn on the left side of the thyroid lead shield and the recorded dose was absorbed by the operator during each case. (**D**) Electronic personal dosimeter screen display. From Abdelaal et al^22^ with permission from Elsevier.

All CCL staff should have their radiation exposure monitored with a dosimeter. Radiation safety officers are responsible for review of dosimeter data so staff may receive feedback on proper dosimeter placement and methods to reduce radiation exposures.^21^ However, standard dosimeters are limited by the lack of real-time updates on cumulative high-grade exposure, and generally, staff may not be notified for weeks to months until their dosimeter is due for review. Development of a real-time radiation level display allows staff to actively react and use radiation reduction strategies to limit exposure. The novel RaySafe i2 system (RaySafe) (Figure 2)^22^ offers real-time x-ray radiation dose monitoring for CCL staff, where the detector badges relay live dose data to a display monitor in the laboratory.^23^ Teams can all see their personal radiation exposure on an overhead screen in the lab and adjust position or make other changes to lower their exposure.

#### Laboratory equipment modifications to reduce exposure

Newer generation angiography systems utilize low-dose imaging technologies. Hardware advancements include improved x-ray tubes, flat panel detectors, spectral beam shaping filters, and pulsed fluoroscopy. Advancements in software have improved image quality, reduced noise at lower radiation dose settings, and avoided unnecessary use of cineangiography.^24^^,^^25^

The generation of x-radiation is reviewed elsewhere and is beyond the scope of this paper. To summarize, energy conversion takes place within the x-ray tube. The quantity (exposure) and quality (spectrum) of the x-radiation produced are automatically adjusting the electrical quantities (kVp, mA) and exposure time applied to the tube. The different x-ray machines on the market have built-in functions to automatically reduce or increase radiation delivery to obtain optimal angiographic images. Furthermore, default protocols for patients of low weight or pediatric age or those undergoing an EP procedure have a tailored approach that utilizes low energy while maintaining high image quality. Operators have the ability to choose from patient specific protocols to optimize image acquisition while limiting radiation exposure.^22^

#### Operator techniques

Recent updates to best practices in radiation safety in the CCL have reiterated important concepts and advances in the technical generation and recording of x-ray imaging. The primary mechanism for radiation exposure to the operator and staff is radiation scatter coming from the patient. Therefore, reducing patient radiation directly reduces radiation scatter to everyone else in the room. The guiding principle of radiation safety is “ALARA,” which stands for “as low as reasonably achievable.” Radiation exposure is dependent on time, distance, and shielding. Operators and staff must maintain good working habits and constant radiation situational awareness to minimize radiation exposure. Techniques that should be implemented include the following:

1. Reduce fluoroscopy time

Activation of the fluoroscopy unit should be minimized by avoiding pressing the fluoroscopy pedal when not looking at the image.

2. Minimize fluoroscopy and cine frame rates

A reduction of the fluoroscopic rate from 15 frames/s to 7.5 frames/s with a low-dose fluoroscopy mode reduces radiation exposure by 67%.^25^ This is especially important for prolonged interventions, such as CTO PCI and some EP cases. The radiation dose of cineangiography image acquisition is also about 6 to 10 times higher than during fluoroscopy.^26^ Therefore, one should minimize the use of cine when possible. Most CCLs now have a “store last fluoroscopy image” (ie, “fluoro-save”) function that can reduce the need for cine and document the different steps of the procedure.^27^

3. Optimize magnification and collimation

Increasing magnification results in increased radiation and should be minimized. Some modern systems allow for magnification without additional radiation (ie, “Live Zoom” feature). This enlarges the image on the field of view without the added radiation.

Using collimators and focusing only on the field of interest helps reduce radiation to the patient and thus reduce radiation scatter. In a room with a large flat panel detector, collimation should be used to focus on the heart for coronary procedures and avoiding “white fields” such as the lungs.^28^ Many systems have integrated dose rate control functions that automatically select exposure parameters for different field exposures by utilizing the shutters. Devices developed to reduce radiation also include the recent Food and Drug Administration-approved ControlRad by Boston Scientific which precisely collimates the area of interest and reduces the dose significantly to peripheral areas in the image.^29^

**Figure 3:**
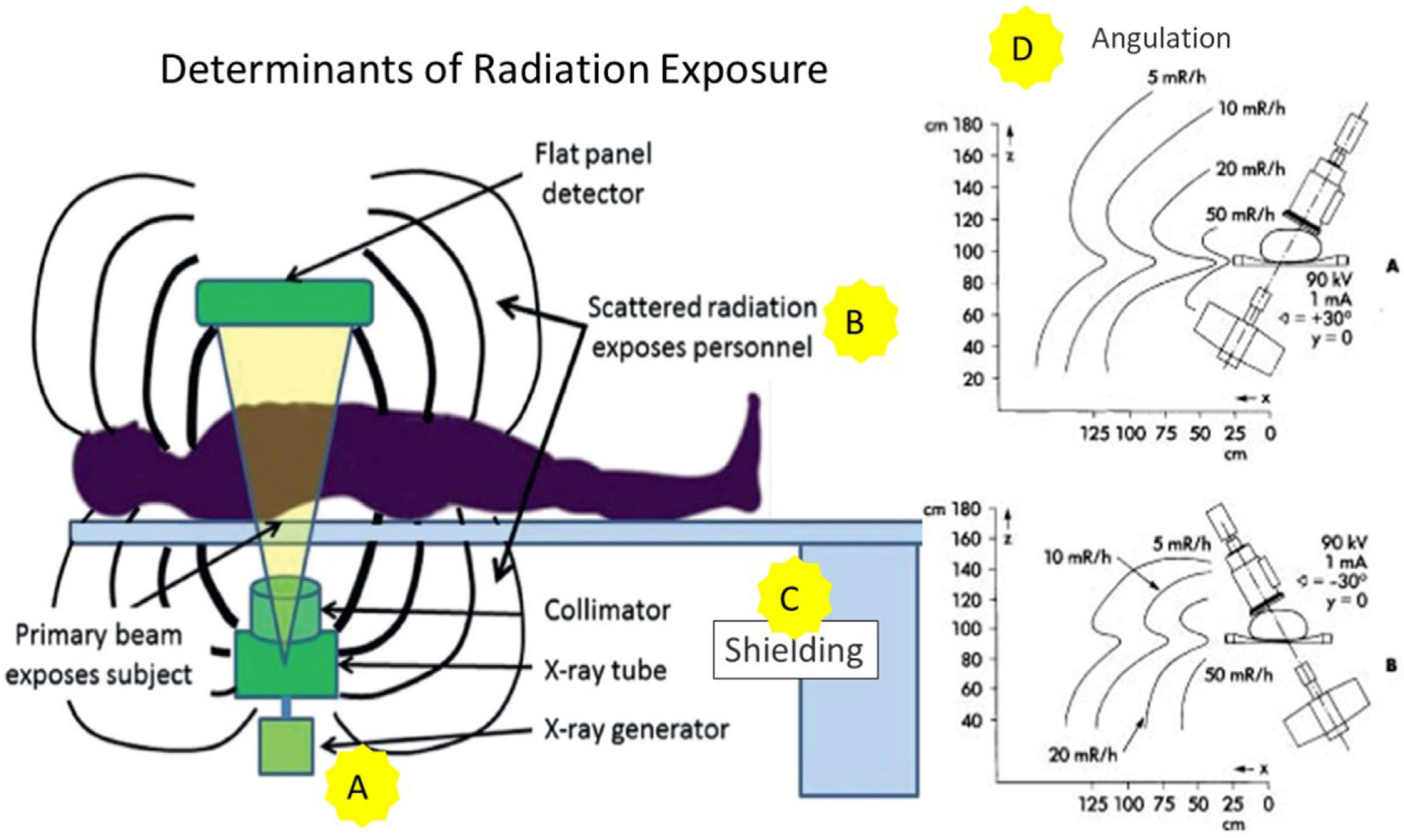
**Diagrammatic Representation of an X-Ray Fluoroscopy System to Illustrate X-Ray Exposure Modality.** (**A**) The primary beam, collimated to a rectangular cross section, enters the patient, typically through the patient’s back. The magnitude of beam exposure can be reduced by minimizing use of magnification, using collimators, decreasing frame rate, and minimizing use of cine. (**B**) Them beam attenuates upon passing through the patient and is scattered within the imaging field. The scattered radiation exposes personnel to radiation. Scatter can be reduced by increasing distance of operator from radiation source, keeping image detector close to the patient, and increasing table height to maximal elevation to increase distance from x-ray generator to the patient. (**C**) Radiation exposure can also be reduced using proper shielding techniques and by (**D**) avoiding steep angulation to reduce scatter. Adapted from Hirshfeld et al^28^ with permission from Elsevier.

4. Employ best techniques for distance, angulation and table position (Figure 3).

Increasing distance from the operator to the radiation source can significantly reduce radiation exposure. The inverse square law for radiation means that doubling the distance from the primary beam and the operator reduces radiation by 4-fold. It is a good practice to use trigger extensions on contrast injectors to allow operators to stand further away from the radiation beam during image acquisition.

**Figure 4:**
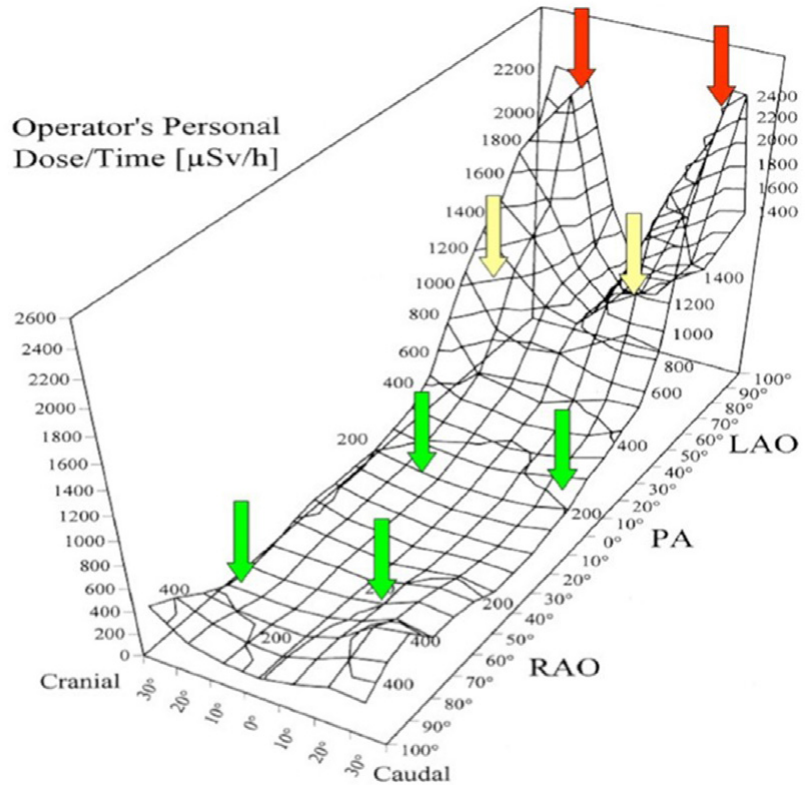
**Radiation dose to the operator**. Calculated dose lines in a three-dimensional graph of the operator’s mean personal dose per time (Sv/h), as a function of tube angulation. LAO, left anterior oblique; PA, posteroanterior; RAO, right anterior oblique. Adapted from Kuon et al^30^ with permission from Elsevier.

Minimizing use of steep angles of the x-ray beam can have a significant decrease in radiation scatter.^30^ Steep angles, such as steep cranial or caudal views, increase the path beam length within the patient resulting in higher radiation scatter and up to a 3-fold increase in radiation dose. The left anterior oblique (LAO) cranial angulation has the highest degree of scatter exposure to the operator on the right side of the patient (Figure 4).

**Figure 5:**
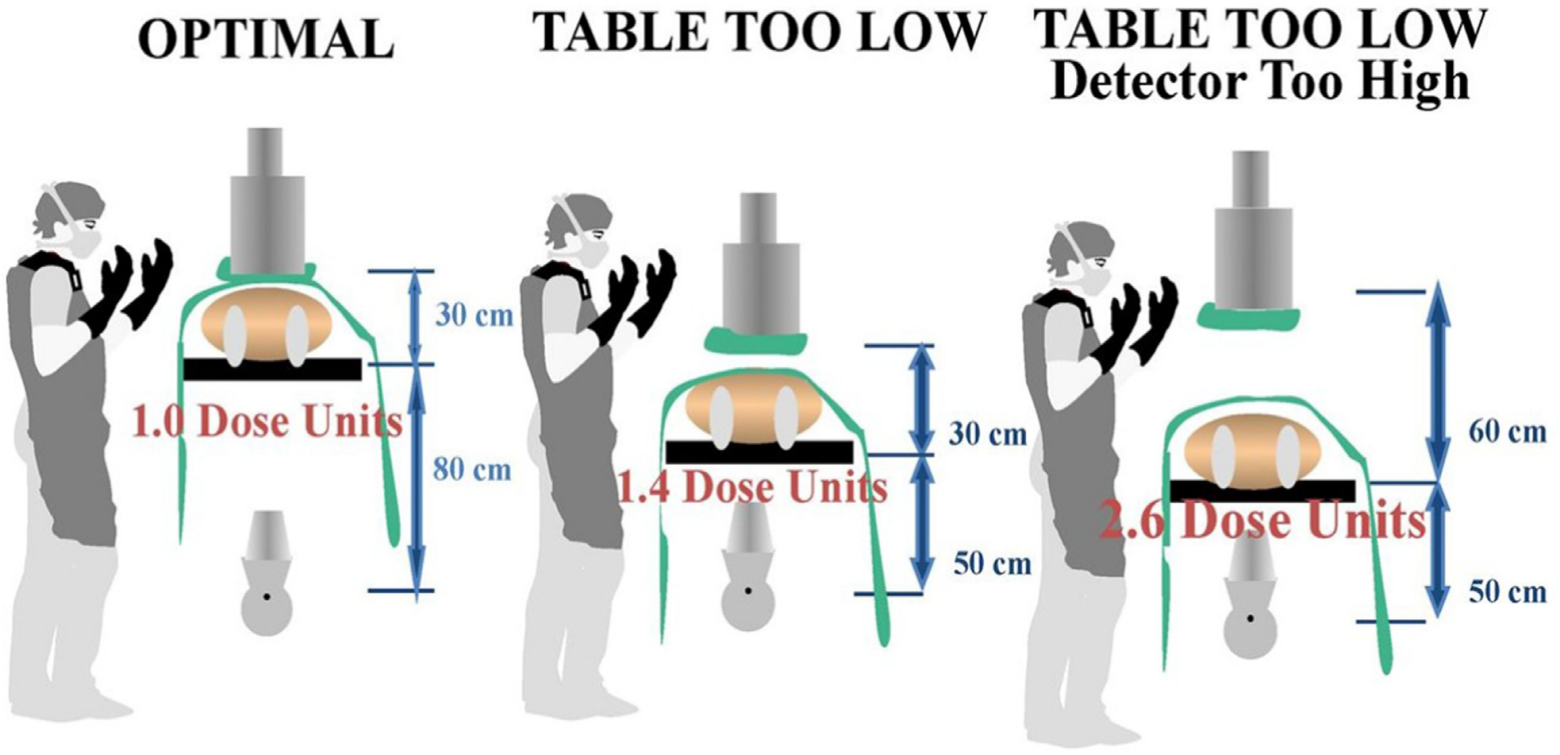
**Diagrammatic Representation of the Effect of System Positioning on Patient and Operator Radiation Exposure During x-ray Fluoroscopy**. Note that in the “table too low” circumstance, the entrance port dose delivered to the patient is increased compared with optimal positioning. In the “table too low, detector too high” circumstance, the entrance port dose to the patient is further increased. In addition, in the “table too low” circumstance, the scattered dose to the operator increases because less of the scattered dose is intercepted by the detector. Adapted from Hirshfeld et al^28^ with permission from Elsevier.

Another precaution to specifically decrease exposure to the operator is to optimize the table height, the distance of the image detector to the patient, and operator position. Methods to reduce exposure to radiation scatter include minimizing the distance between the image detector and the patient (low subject–image distance) and maximizing the table height from the x-ray tube while still maintaining operator comfort (Figure 5).

#### Radiation exposure with radial versus femoral intervention

Coronary angiography and PCI via radial access has continued to grow in use worldwide. Although data has been mixed, some publications suggest that transradial access may be associated with higher radiation.^31^, ^32^, ^33^ Interestingly, data from the French multicenter RAY'ACT-1 study showed that radial access was actually associated with lower radiation than femoral access in high volume centers.^34^ A number of variables influence radiation exposure with radial access, with the most notable being institutional and operator familiarity and volume, patient characteristics and comorbidities, laterality (eg, right vs left radial access), and equipment or catheter selection.^33^^,^^35^^,^^36^

A meta-analysis of randomized controlled trials reporting primary outcomes of fluoroscopy time and dose-area product between transradial and transfemoral approaches between the years 2014 and 2021 showed that although the radial approach was associated with increased radiation exposure, the gap has decreased from year to year with crossover around the year 2019.^33^ As radial access operators’ competency increases, the decision to perform radial or femoral access will not be made based upon radiation exposure but rather on patient and procedural needs. Choosing one access approach over another likely does not mitigate radiation dose exposure. Nonetheless, this emphasizes the overall importance of reducing radiation exposure for all cases in the CCL.

#### Removing the operators from the environment: robotic-assisted interventions

**Figure 6:**
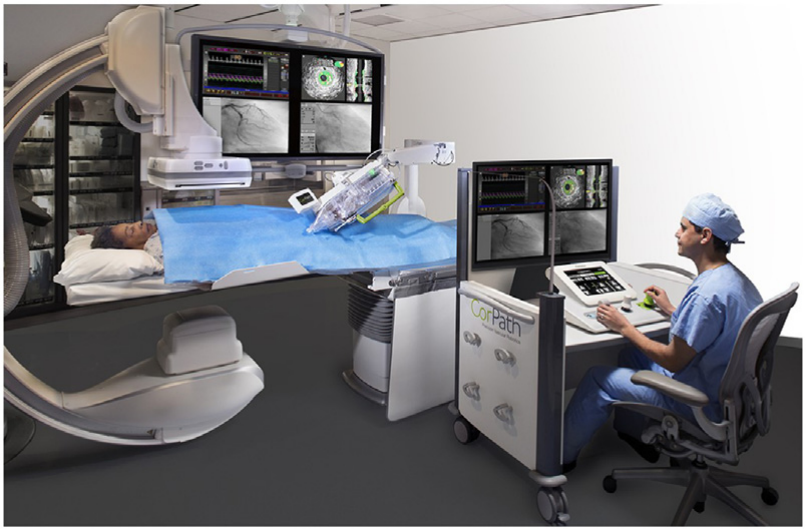
**The Corindus CorPath uses a robotic system that relocates the operator from bedside to a remote console to perform procedures from a distance**. Corpath GRX robotic system for use during percutaneous coronary interventions. Reprinted with permission from Siemens Medical Solutions Inc.

The Corindus CorPath (Siemens Medical Solutions USA, Inc) is a Food and Drug Administration-approved technology designed to relocate the operator from bedside to a remote console permitting the operators to perform procedures from a distance—dramatically reducing exposure and ergonomic hazards (Figure 6). Using a robotic system allows operators to shed their lead aprons and sit in a lead-lined booth away from the radiation field to remotely control catheters.^37^

The large-scale multicenter PRECISE (Percutaneous Robotically-Enhanced Coronary Intervention) study of the Corindus CorPath robotic PCI system showed 98.8% technical success rate without device-related complications and 97.6% clinical procedural success with 2.4% having periprocedural non–Q-wave myocardial infarctions. Although radiation exposure was not a clinical end point, there was a median reduction of radiation exposure of 95.2% as well as the benefit of spending a significant portion of the procedure sitting in the console.^38^ Without the need for lead aprons, this minimizes risk of orthopedic or musculoskeletal injury.

As procedural complexity in coronary intervention grows, robotic-assisted percutaneous intervention becomes increasingly valuable for its ability to reduce operator occupational exposure. CORA-PCI (Complex Robotically Assisted Percutaneous Coronary Intervention) demonstrated technical success rate of robotic PCI to be comparable to manual PCI (*P* = 1.00) for more complex cases, excluding atherectomy, planned 2-stent bifurcation lesions or CTO where a hybrid robotic-manual approach is required. CORA-PCI showed a significant reduction in dose-area product (cGy∗cm^2^) in the robotic versus manual PCI groups (*P* = 0.045), although overall fluoroscopy time was not significantly different (*P* = 0.39).^39^ The Robotic-Assisted Peripheral Intervention for peripheral arterial Disease study demonstrated feasibility of robotic peripheral vascular interventions. The small single-arm study enrolled 20 subjects and evaluated device technical success (defined as successful cannulation of the target vessel using the CorPath 200 system), device safety (defined as absence of device-related serious adverse events), and clinical procedural success (< 50% residual stenosis without unplanned manual conversion or assistance or periprocedural device-related adverse events). RAPID demonstrated similar fluoroscopy time (7.1 ± 3.2 min) and contrast use 73.3 ± 9.2 mL) to manually performed peripheral cases in similar patient cohorts.^40^

Although robotic-assisted intervention is a promising approach to reduce radiation exposure for operators, it does not confer immediate protection for the rest of the CCL team. The potential benefits of robotics and automation of other CCL staff roles should be further explored.

### Novel methods for radiation protection

**Figure 7:**
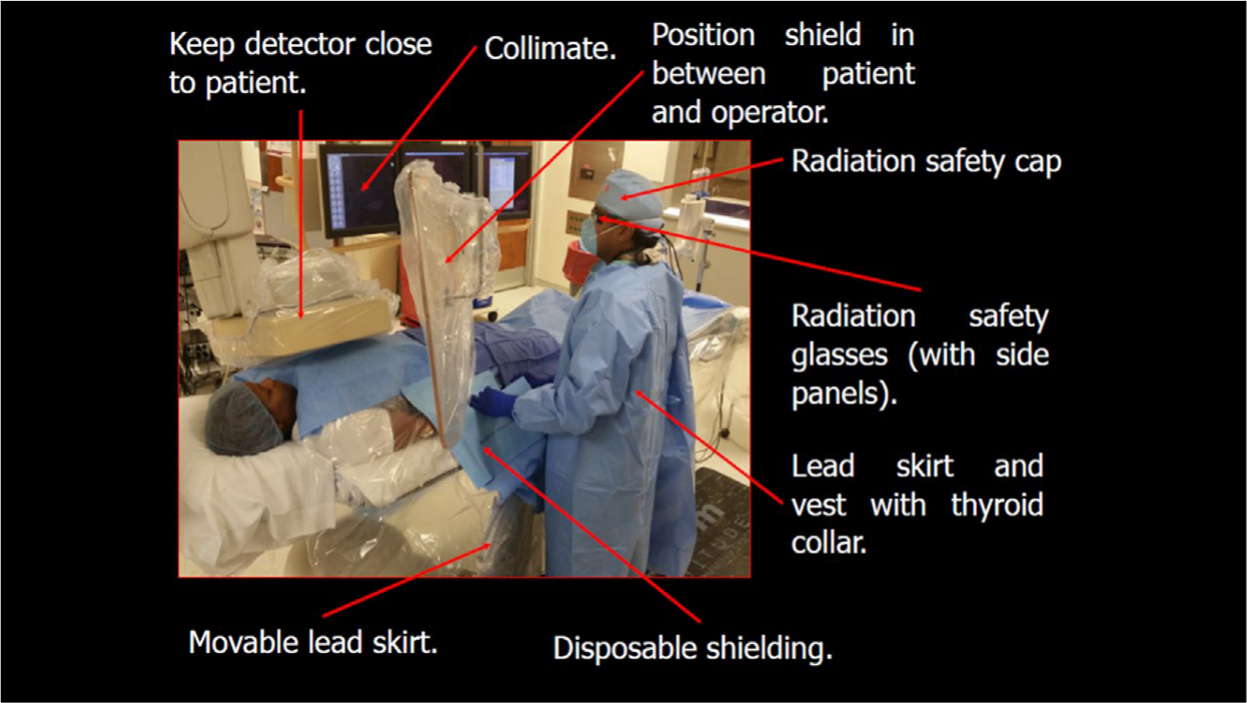
**Current best practices to minimize radiation exposure to the patient and operator in the cardiac catheterization laboratory**. From Kumar and Tanveer Rab.^41^

Several technologies are now available that can markedly reduce scatter radiation, and even remove the operator from the radiation field altogether. These approaches may allow CCLs to achieve the goal of removing lead aprons altogether. Figure 7 provides an overview of potential areas to reduce radiation.^41^

#### Thyroid shielding

The association between radiation exposure and risk of thyroid cancer has been well established. The risk has been shown to be proportional to cumulative dose exposure and age at exposure, with greater risk at younger age, particularly less than 20 years of age.^42^

Limited studies have compared thyroid shield designs and radiation exposure. One study showed that a properly fitted thyroid collar with a bismuth masking reagent compared to a collar that was too tight or too loose on the neck resulted in the lowest radiation exposure in μSv/min during C-arm fluoroscopy.^43^ Studies of radiation exposure in dental panoramic imaging demonstrated that thyroid collar design affects penetrating radiation dose. Effective shielding area and material composition are the most important factors in reducing exposure; effective shielding areas of at least ∼ 300 cm^2^ are recommended. Nominal thickness is less important, although the standard is 0.25 to 0.5 mm thickness of lead.^44^

#### Eye protection

The goal of protective eyewear is to provide maximal shielding from front, lateral, and angular radiation while maintaining good vision and reducing eye fatigue. Studies comparing radioprotective eyewear have demonstrated that optimal thickness is 0.35 mm to 0.5 mm of lead glass. The gap between the lens and frame of the radioprotective eyewear and the length of the front radioprotective glass contribute significantly to angular protective shielding.^45^ Materials used in eyewear include Kynetium, Grilamid, titanium, and carbon fiber with clear lead protective lenses. Anti-reflective coatings and anti-fog coatings are also featured on some designs. Proper fit and facial contour are important for reducing penetrating radiation exposure. Special designs have come to market intended for individuals with certain facial features such as flatter nasal bridges.

#### Patient-applied radiation shields

A simple lead apron applied on the patient is a feasible, practical, and inexpensive method. Pelvic lead shielding of the patient has been reported to reduce radiation exposure significantly for the operator, during cardiac catheterization in both femoral and radial approaches.

**Figure 8:**
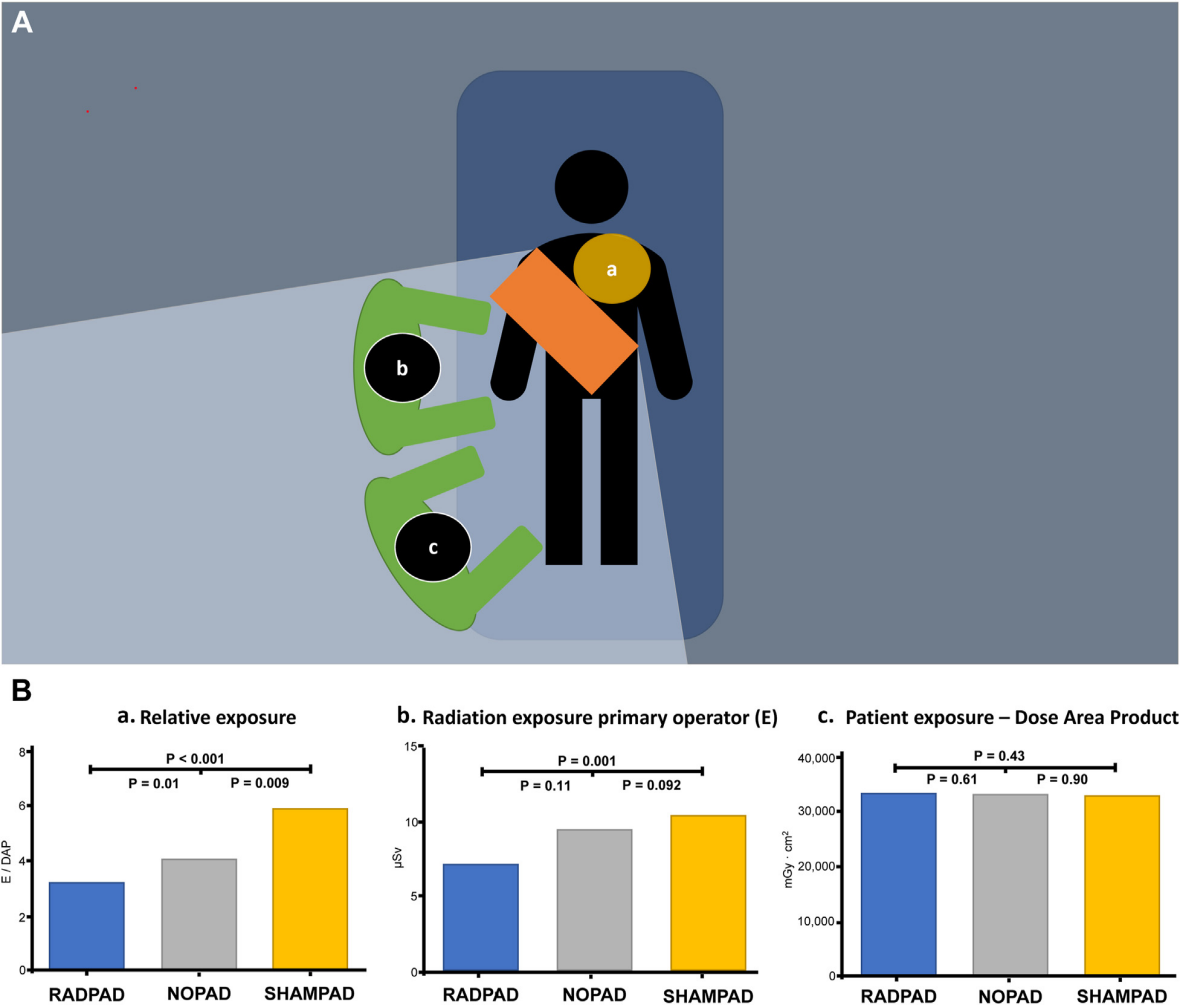
(**A**) **Position of the absorbing shield, placed on the patient, between the image intensifier**. (a), the primary operator (b), and occasionally the secondary operator (c). (**B)** Relative exposure. (a) (×10^–4^) is defined as the ratio between the exposure of the primary operator at chest level (b) and the patient exposure per procedure (c). Data are presented as mean (A) or median (B and C). DAP indicates dose-area product; NOPAD, standard treatment; RADPAD, radiation absorbing shield; and SHAMPAD, sham shield. Data from Vlastra et al.^46^

Disposable radiation shielding pads such as the RADPAD (Worldwide Innovations & Technologies, Inc) are sterile, disposable, lead-free shields placed on the patient between the image intensifier and the operator, that have been shown to significantly reduce radiation exposure to the operator in multiple trials (Figure 8).^46^, ^47^, ^48^, ^49^ However, these methods do involve additional equipment and cost.

#### Novel radiation shielding for a “lead-free” environment

Innovative products that reduce both exposure and orthopedic injury include a floor or ceiling-suspended body shielding unit, protective radiation cabins, or improved systems to reduce scatter. Lastly, specific arrangements of shielding that surround the patient and tube now permit operators to work without personal protective equipment. These novel approaches to radiation shielding require the acquisition of additional equipment and increased costs, limiting integration of these technologies to many facilities.

The Zero-Gravity system (BIOTRONIK) is a 1-mm lead body shield that is suspended either from a floor unit or from the ceiling (Central Illustration). It has been shown to significantly reduce radiation exposure for the operator while minimizing the weight carried by the operator.^50^

**Central Illustration:**
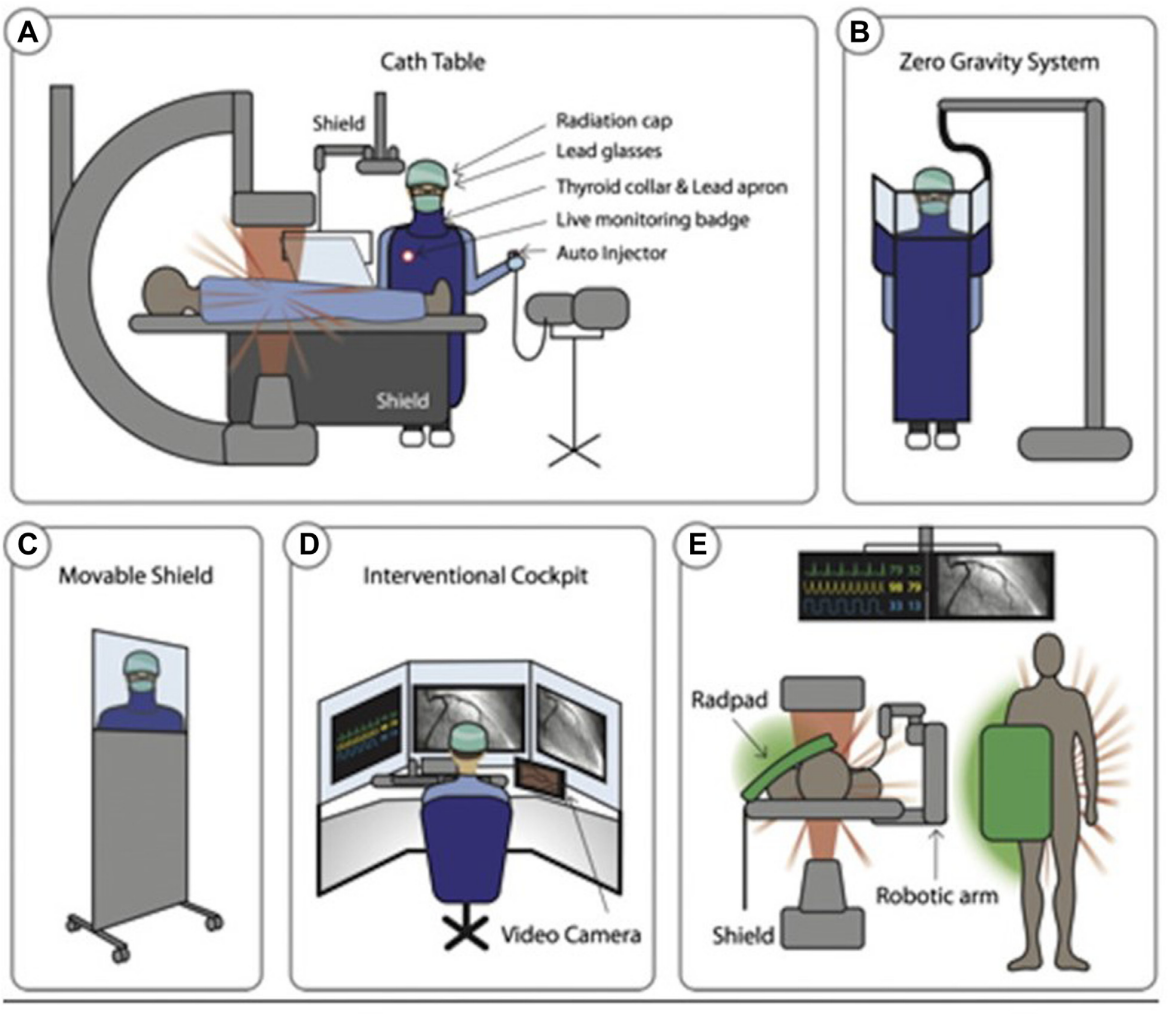
**Novel technologies with potential to further reduce radiation exposure.** (**A**) The current best practices in the catheterization lab is depicted. Note that a live monitor badge may be used for real-time information of radiation exposure. (**B**) The Zero-Gravity System has been shown to significantly reduce radiation exposure for the operator while minimizing the weight carried by the operator. (**C**) A mobile side shield can be useful to protect medical personnel from radiation to the right side of the patient. (**D**) The application of robotic-assisted interventions moves the proceduralist from the bedside to a ​remote console which dramatically reduces exposure hazards. (**E**) Disposable radiation shielding pads, such as the RADPAD, can minimize scatter radiation to the operator. Reprinted from Abuzeid et al^48^ with permission from Elsevier.

Radiation protection cabins, such as the Cathpax cabin (Lemer Pax), are glass walled structures with openings for the operator to access the sterile field that have also been shown in trials to reduce radiation exposure.^51^

Unique patient-centered radiation shielding systems are also available. The Radiaction system (Radiaction Medical) is a robotic radiation shielding system that was developed to provide full body protection to all medical personnel during fluoroscopy-guided procedures by “encapsulating” the imaging beam. This aims to block scattered radiation. Preliminary phantom and clinical evaluation demonstrated that the system is safe and easily integrated into the clinical workflow (Central Illustration).^52^

**Figure 9:**
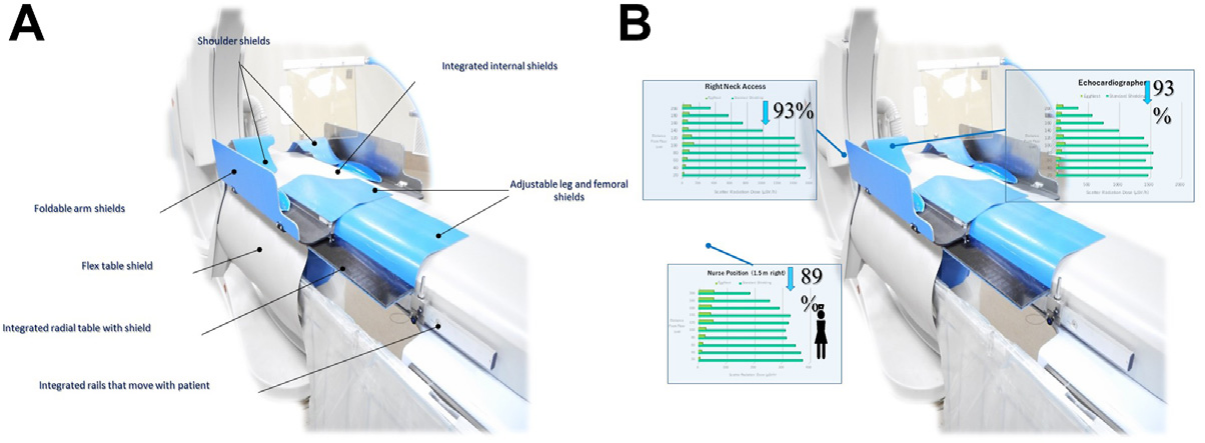
(**A**) **EggNest-XR System**. (**B**) Effect of EggNest on scatter radiation: head and nurse positions. Percentages compared to standard shielding. Courtesy of Dr. Robert Wilson.

The EggNest-XR system (Egg Medical) is comprised of a carbon fiber base platform with integrated mattress, rail systems, arm board, and shielding components including multiple flexible and flip shields (Figure 9). These shields can be adjusted to conform to the patient’s body to reduce scatter. Preliminary data suggests the system produces an average of 91% reduction in total room scatter radiation when compared to conventional shielding.^53^ Significant reduction in radiation dose has been reported for multiple standard camera angles and for different methods of access including neck and radial access. The system has not yet been evaluated in large clinical studies.

A novel vertical radiation shield system (Steradian) has been demonstrated to reduce operator radiation exposure. Using phantom models and clinical dosimeter studies, Panetta et al^54^ found that operator exposure was significantly reduced by utilizing the vertical shield coupled with increasing distance from the x-ray tube, using lower magnification, and avoiding LAO-caudal angles.

**Figure 10:**
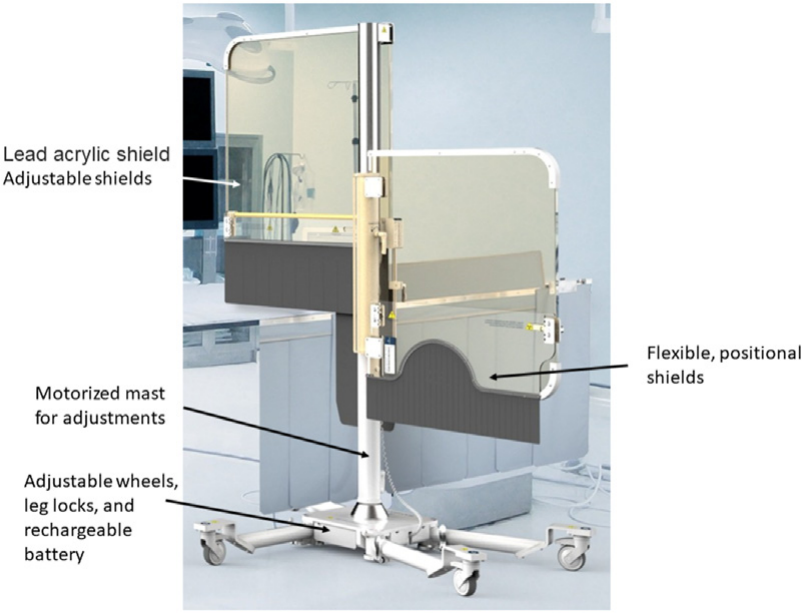
**The RAMPART M1128 radiation shielding system is a mobile lead radiation protection devices that is designed specifically to allow operators to forgo wearing lead altogether**. Reprinted with permission from RAMPART ic, LLC.

Mobile lead radiation protection devices, such as the Rampart M1128 device (Rampart ic, LLC), were designed specifically to allow operators to forgo wearing lead altogether (Figure 10). The device is comprised of a configurable, floor-mounted center mast that supports 2 thick lead panels above the table and 2 lead curtains below the table. The lead panels are each 1-mm thick and are attached to either side of a center mast. Accessory soft lead shielding that are 0.5-mm thick attach to these lead panels and cover the patient. The device is positioned over the patient’s torso and can be angled at 180-degree configuration for structural and bi-plane set-up or at 90-degree configuration for EP and standard or complex coronary interventions. Of note, there is lack of protection for personnel at the head of the bed and the left side of the table. There is an ongoing clinical study to compare the efficacy of this device to conventional systems.^55^

**Figure 11:**
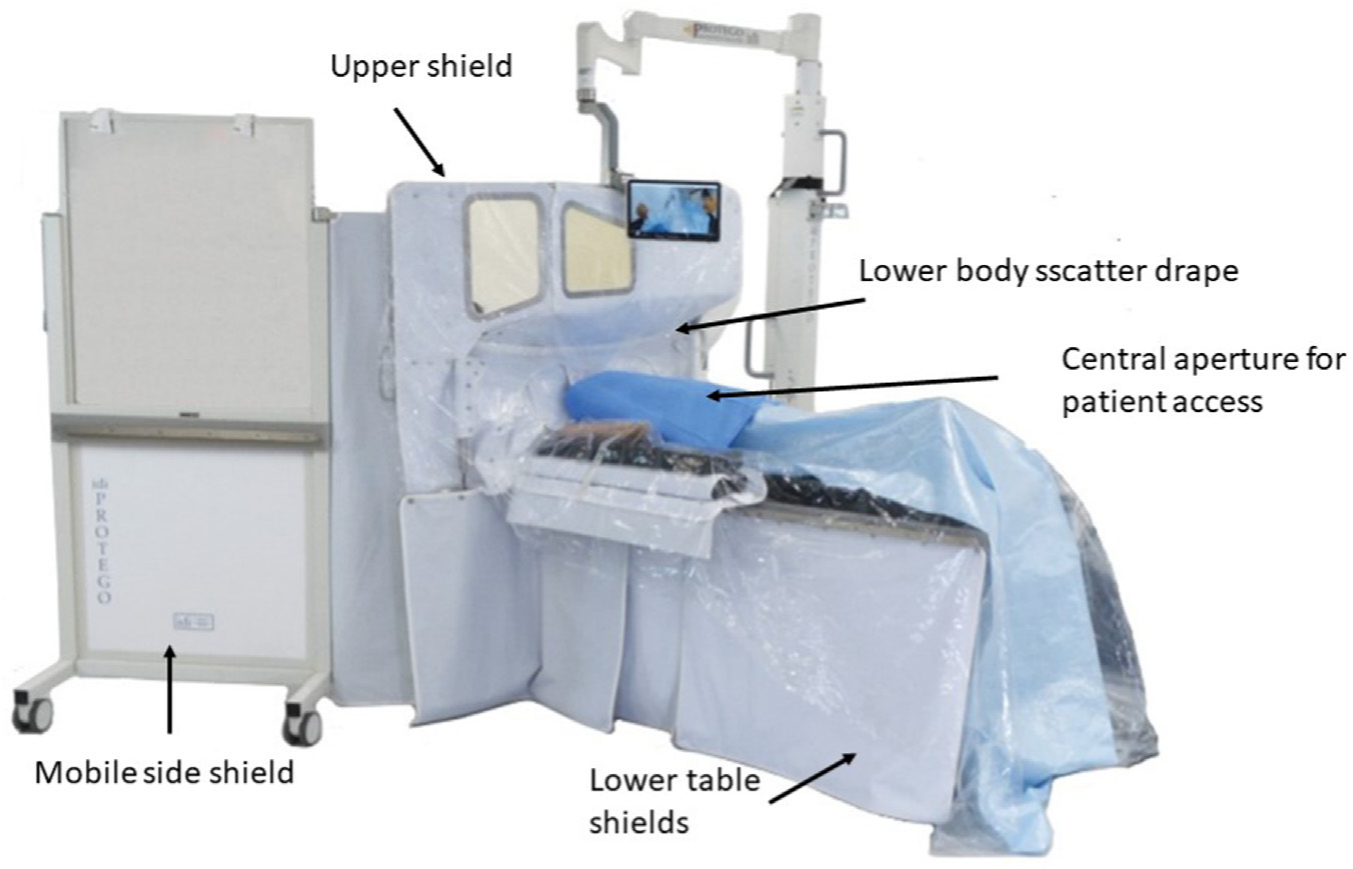
**The Protego Radiation Protection System incorporates concepts of reducing scatter radiation with patient shield pads, lower table shields, angled radiation barrier wall, and a mobile side shield**. Reprinted from Allen et al.^56^

The Protego Radiation Protection System (Image Diagnostics, Inc) incorporates concepts of reducing scatter radiation with patient shield pads as well as lower table shields (Figure 11).^56^ It also includes an angled radiation barrier wall sitting between the imaging equipment and the health care personnel and a mobile side shield to the right of the table. A preclinical study using a scatter radiation phantom demonstrated a >94.2% reduction in scatter radiation across 20 reference points on the operator side of the table as well as dose reduction at the location of the primary operator ranging from 97.8% to 99.8% in posteroanterior and LAO projections.^57^

## Conclusion

As long as ionizing radiation is required for invasive cardiology procedures, radiation protection will continue to evolve. Novel innovations in personal protection as well as patient-centered room shielding will significantly reduce exposure. It is a common goal to apply best practices to reduce radiation exposure and embrace proven technologies leading to a more efficient, safer, and comfortable lead-free working environment.
